# Metronomic chemotherapy and drug repurposing: A paradigm shift in oncology

**DOI:** 10.1016/j.heliyon.2024.e24670

**Published:** 2024-01-14

**Authors:** Nusrat Jan, Shazia Sofi, Hina Qayoom, Aisha Shabir, Burhan Ul Haq, Muzaffar A. Macha, Abdullah Almilaibary, Manzoor Ahmad Mir

**Affiliations:** a Department of Bioresources, School of Biological Sciences, University of Kashmir, Srinagar-190006, India; b Watson-Crick Centre for Molecular Medicine, Islamic University of Science and Technology, Pulwama, India; c Department of Family and Community Medicine, Faculty of Medicine, Al Baha University, Saudi Arabia

**Keywords:** Metronomic chemotherapy, Conventional chemotherapy, Drug repurposing, Breast cancer, Drug resistance, Angiogenesis

## Abstract

Cancer represents a significant global health and economic burden due to its high mortality rates. While effective in some instances, traditional chemotherapy often falls short of entirely eradicating various types of cancer. It can cause severe side effects due to harm to healthy cells. Two therapeutic approaches have risen to the forefront to address these limitations: metronomic chemotherapy (MCT) and drug repurposing. Metronomic chemotherapy is an innovative approach that breaks from traditional models. It involves the administration of chemotherapeutic regimens at lower doses, without long drug-free intervals that have previously been a hallmark of such treatments. This method offers a significant reduction in side effects and improved disease management. Simultaneously, drug repurposing has gained considerable attraction in cancer treatment. This approach involves utilizing existing drugs, initially developed for other therapeutic purposes, as potential cancer treatments. The application of known drugs in a new context accelerates the timeline from laboratory to patient due to pre-existing safety and dosage data. The intersection of these two strategies gives rise to a novel therapeutic approach named ‘Metronomics.’ This approach encapsulates the benefits of both MCT and drug repurposing, leading to reduced toxicity, potential for oral administration, improved patient quality of life, accelerated clinical implementation, and enhanced affordability. Numerous clinical studies have endorsed the efficacy of metronomic chemotherapy with tolerable side effects, underlining the potential of Metronomics in better cancer management, particularly in low- and middle-income countries. This review underscores the benefits and applications of metronomic chemotherapy and drug repurposing, specifically in the context of breast cancer, showcasing the promising results of pre-clinical and clinical studies. However, we acknowledge the necessity of additional clinical investigations to definitively establish the role of metronomic chemotherapy in conjunction with other treatments in comprehensive cancer management.

## Highlights

• Cancer, a leading cause of mortality, is traditionally treated with chemotherapy, which faces significant drawbacks, including high doses, side effects, and the risk of multiple drug resistance.
• Metronomic chemotherapy (MCT) and drug repurposing are 21st-century approaches developed to tackle the limitations of traditional chemotherapy.
• Drug repurposing expands treatment options using existing drugs, offering attractive attributes shared with MCT, such as approved drug usage, fewer side effects, and improved patient quality of life.
• Our review explores the potential of MCT and drug repurposing in managing cancers, including breast cancer, reviewing their mechanisms of action and analyzing pre-clinical and clinical trials.

## Graphical abstract

**Figure:**
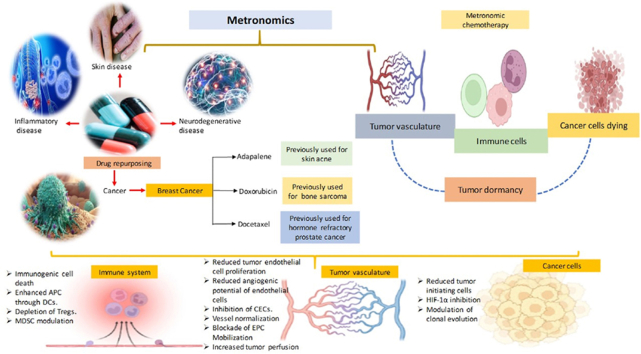

## 1 Introduction

Globally after heart disease, cancer is the 2nd main reason for mortality and a significant public health issue [1,2]. According to a prediction by Global demographic characteristics, there will be an elevation in the incidence of cancer in the next decades, with more than twenty million new cancer cases annually expected by 2025. In 2018, 9.6 million deaths were estimated and in women worldwide GLOBOCAN predicted 24.2 % of cancer incidence including a death rate of fifteen percent [3]. Breast cancer (BC), particularly has a significant occurrence and mortality rate [[4], [5]], making it the most widespread form of cancer among women globally. It is a type of cancer connected to a substantial number of deaths[6]. In 2020, BC accounts for almost 685,000 deaths in women globally [7]. The primary reason behind this elevated mortality is the emergence of drug resistance, which poses a major obstacle in the treatment of BC [8]. Prevention, early detection, and total eradication are the three crucial priorities when dealing with cancer disease [[9], [10]]. Depending on the disease stage and subtype classification, breast cancer treatment typically employs a multi-modal treatment approach using combinations of surgery, chemotherapy, endocrine therapy, and radiotherapy. The mainstay of therapy is, however, chemotherapy combined with surgery, particularly in advanced stages of the disease [[11], [12]].

A German chemist Paul Ehrlich introduced the term chemotherapy and also explored the application of pharmaceutics to cure infectious disorders. In cancer management, recurrence of cancer and metastasis occurred due to radiation therapy and surgery while combinational therapy showed good results [13]. Chemotherapy is thought to be the most effective and widely used treatment for cancer, whether it is given alone or in conjunction with radiation therapy. In recent times, there have been notable advancements in the treatment of carcinoma, as various therapeutic approaches and their specific mechanisms have shown significant progress in combating the disease. Multiple targeted modalities or traditional chemotherapeutics such as taxanes and platinum compounds were used in combination and were found to have synergistic effects. Additionally, new therapeutic approaches are used to treat illnesses, including biological molecules, medications, and immune-based therapies [[14], [15], [16]]. Traditional chemotherapy medicines have different administration regimens, and toxicity profiles, including antitumor activity from molecularly targeted therapies [17]. Traditional chemotherapy does have certain advantages, but there are also significant drawbacks, including a requirement of high doses, poor absorption, side effects, minimal therapy indices, non-specific targeting, and the emergence of multiple drug resistance (MDR) [18]. Even modern drug delivery methods cause undesirable therapeutic reactions that limit dosage and impair the effectiveness of anti-neoplastic medications. Therefore, finding novel therapeutic targets including new treatment strategies is crucial in enhancing cancer treatment. During the 21st century, novel therapeutic approaches called “metronomic chemotherapy” (MCT) and drug repurposing also called drug repositioning has been developed to address the limitations of traditional chemotherapy [19].

The Metronomic chemotherapy (MCT) treatment was introduced by Kerbel [17] and Hanahan in 2000 [20]. The researchers J. Folkman and R. Kerbel groups established the framework for this therapeutic approach. In various experimental models, researchers investigated the knowledge of drug effects for various tumor types, the mechanism of action, and appropriate therapeutics for their use in MCT schedules both for individual or combined treatments [21]. It is a novel and effective treatment method for malignant and blood malignancies and involves the continuous administration of chemotherapy at low doses, which allows for sustained and active levels of medicines in the bloodstream for extended periods and has demonstrated positive tolerability and favorable outcomes. The main mechanism of action is not a direct antitumor effect. MCT mostly affects tumor cells indirectly, particularly in their microenvironment by preventing tumor angiogenesis. It also promotes the host's immunological response to cancer and affects stromal tissue [22].

Recent preclinical investigations have revealed that repeated application of the minimal amount of several anti-tumor medications, known as “metronomic” chemotherapy, at doses ranging from one-tenth to one-third of the maximum tolerated dose (MTD), increases the anti-angiogenic properties of the medication. Concurrent administration of a different agent that slows growth and tumor formation may further enhance the benefits of these metronomic regimens of anti-tumor drugs. Metronomic chemotherapy is used for prolonged periods to minimize side effects and effectively target both endothelial cells and neoplastic cells that are in the proliferative stage [23]. It was previously believed that they act antiangiogenically, but more recently, new modes of action have been investigated, establishing MCT as a multi-targeted therapy. Today, MCT is seen as a viable choice for patients who have advanced to many lines of chemotherapy and, more critically, for pediatric cancer in low and middle-income countries (LMICs) [24,25].

**Fig. 1:**
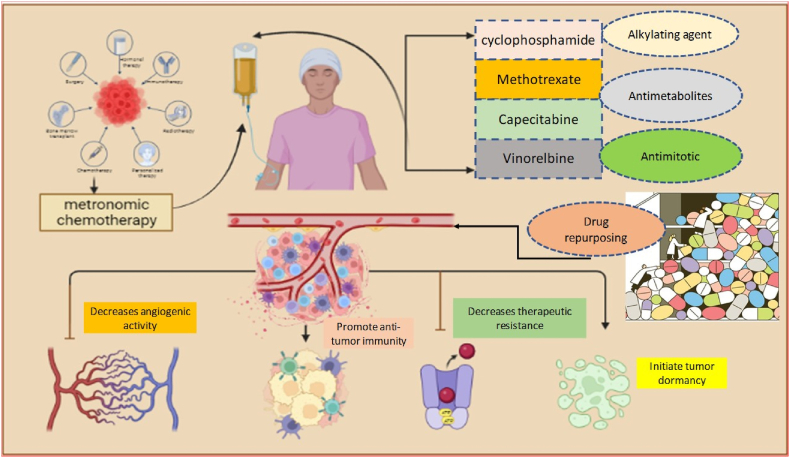
Combinatorial role of metronomic chemotherapy and drug repurposing in initiating the anti-tumor activity via several mechanisms.

After the MCT, the idea of drug repurposing also called drug repositioning gained strength in cancer treatments. Drug repositioning is a strategy of finding a new use for already-existing drugs that were used for other ailments and can be now used in the treatment of cancer [26,27]. It is an attractive strategy that aims to increase the range of treatment resources without the usage of new pharmacologic advancements, instead making use of those that are currently available. The advancement in understanding the impact of various therapeutics on signaling pathways, metabolism, and gene expression including the relationship between biological activity and chemical structure predicted the significance of these old drugs in cancer treatment. This approach is attracting the attention of many researchers because the information regarding the side effects, pharmacodynamics, toxicities profiles, interaction with other drugs, and pharmacokinetics has been collected and is already known. Further, this approach considerably allows low costs and reduces the time needed to convert it to the clinic until the drug is approved than developing a drug de novo [4,28] Fig. 1.

Soon after the two approaches MCT and drug repurposing were combined and given the name “metronomics” [29]. Both strategies share several characteristics that make “metronomics” an appealing choice for the treatment of cancer, such as the use of approved and known drugs, which reduces the time needed to enter the clinic, including oral administration, fewer side effects, low toxicity, low costs due to the use of generally out-of-patent drugs, a better quality of life and last but not least, the potential for use in nations with very limited economic resources. Here in this review, we looked into the preclinical and clinical experience with low-dose MCT and drug repurposing, highlighting the potential contribution of this novel therapeutic approach to evolving paradigms in the systemic management of breast cancer patients.

## 2 Metronomic chemotherapy (MCT)

MCT is a method of administering cytotoxic drugs that is distinct from traditional chemotherapy schedules. Conventional therapy is based on the administration of maximum doses of chemotherapy drugs, whereas MCT involves the continuous application of chemotherapeutics at low doses that are significantly below the MTD, without significant time gaps between doses [20,30,31]. Initially, it was thought that metronomic chemotherapy's (MCT's) mode of action involved inhibiting angiogenesis. But it is now generally acknowledged that MCT has a variety of mechanisms, including anti-angiogenic, anti-proliferative, and immunomodulatory properties [30,[32], [33], [34], [35]]. The primary features of metronomic chemotherapy include frequent uninterrupted administration of chemotherapy (dose-dense); Use of a biologically optimized dose in place of MTD; low rate of side effects related to treatment; preference for oral medications; potential to develop delayed resistance [36]. As it provides a good balance between efficacy and side effects of the treatment, it may increase the therapeutic index of medications. It also allows for prolonged treatment, which probably increases survival. Because MCT requires regular drug delivery, oral medicines are a more suitable choice for patients [37].

Numerous investigations have so far shown that the majority of MCT-based clinical trials were effectively tolerated. In addition, as evaluated in multiple clinical trials, this therapeutic approach is regarded as a cost-benefit treatment choice. Metronomic chemotherapy has been linked to low toxicity, and major adverse events are uncommon in terms of side effects. The severe toxic consequences are either hardly ever observed or extremely uncommon. In general, side symptoms included nausea, minor-grade fatigue, mild to severe leucopenia, lymphopenia, neutropenia, and anemia [38,39].

Based on research by Browder et al. Klement et al. and Hanahan et al. originally suggested the word “metronomic” in 2000 and sparked the scientific community's interest in further examining this treatment plan in preclinical and clinical investigations to determine its efficacy and methods of action [[40], [41], [42]]. The Folkman and Kerbel laboratories were the first to conduct the initial preclinical research on metronomic chemotherapy. Browder and colleagues developed an animal xenograft model using low-dose metronomic (LDM) treatment in research conducted in the year 2000. Mice with Lewis lung cancer that was resistant to the MTD of cyclophosphamide was given the same medication on a metronomic schedule every six day. Cyclophosphamide demonstrated a notable reduction in tumor growth when used in the LDM therapy regimen [41].

A follow-up study conducted in the same year by Klement and colleagues also verified that the antiangiogenic drug DC101, an antibody that blocks the vascular endothelial growth factor receptor 2 (VEGFR2), is only effective in combination with continuous administration of vinblastine in a LDM chemotherapy regimen. This combination was found to extend the anti-tumor activity of neuroblastoma tumors [42]. The long-term low-dose vinblastine reduced tumor angiogenesis, causing massive, well-established tumors to shrink. Following these investigations, the impact of metronomic chemotherapy (MCT) when combined with various medications has been gradually investigated [42,43].

According to a PubMed database, numerous studies on the preclinical effects of MCT have been published so far. These publications discuss the therapeutic effectiveness of MCT in treating different tumors [31]. Cyclophosphamide is the most frequently used anticancer drug for preclinical MCT research. The powerful anticancer effectiveness of MCT regimens in models of advanced metastatic cancer is an intriguing finding in several of these trials, especially when paired with a specific antiangiogenic drug that has low activity by itself in this setting [44,45].

Many randomized phase III trials have been launched, considering the excellent outcomes observed in preclinical and clinical studies investigating MCT alone or MCT in combination with targeted agents, particularly antiangiogenic drugs. These trials encompass various types of carcinomas including BC, aiming to further evaluate the efficacy and potential benefits of MCT and its combinations [46]. The antiangiogenic drugs, when used, are bevacizumab (Avastin), the monoclonal anti-VEGF antibody and the chemotherapeutics involved are cyclophosphamide (CTX), methotrexate (MTX), and capecitabine (CPB).

## 3 Advantages of metronomic chemotherapy over conventional chemotherapy

**Fig. 2:**
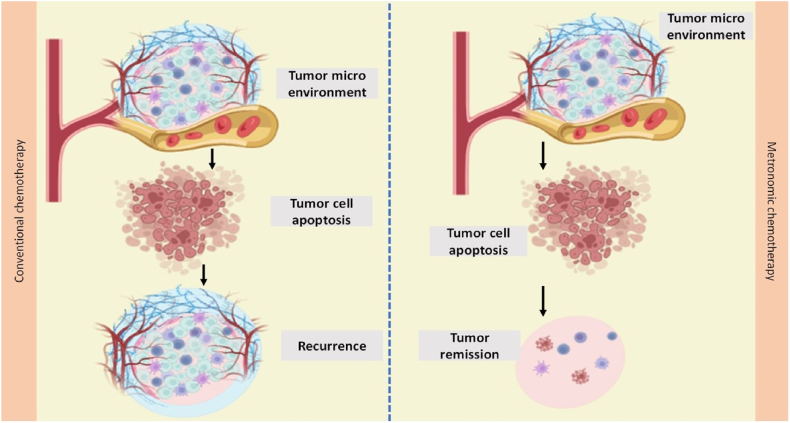
Representation of how metronomic chemotherapy differs from conventional chemotherapy. Conventional chemotherapy is always associated with tumor recurrence as compared to the metronomic chemotherapy where tumor recurrence rate is severely low.

Despite conventional chemotherapy has generally high efficacy but still has certain drawbacks which include several side effects and a weakened immune system's ability to fight the tumor. Metronomic chemotherapy (MCT) is a new therapeutic approach that has been created to target tumors and combat their adverse consequences [47,48]. This new therapeutic approach to chemotherapy administration entails administering cytotoxic drugs at lower doses more frequently [34]. Metronomic chemotherapy (MCT) has many therapeutic advantages over conventional chemotherapy such as in terms of dosage and frequency of drug administration, reduced tumor vascularization, decreased drug resistance, pharmacokinetics, cellular and molecular targets, host toxicity, and most importantly, enhanced anti-tumor immune responses and patients quality of life [49,50]. Both regimens have different therapeutic targets in which conventional chemotherapy predominantly targets tumor cells that divide quickly while the angiogenically stimulated endothelial cells already present in tumor blood vessels are the main targets of MCT [50]. MCT is an advanced option to conventional chemotherapy. Additionally, its lower price, proper toxicity profile, and ease of application make it superior to conventional treatments. Table 1 illustrates the general distinction between metronomic and conventional chemotherapy [51,52] Fig. 2.

**Table 1:** Distinguishing features between conventional chemotherapy and metronomic chemotherapy.

S.no.	Parameters	Traditional/Conventional chemotherapy	Metronomic chemotherapy (MCT)	References
1	Dose	Developed to deliver at a maximum tolerated dose (MTD)	Low doses below the MTD	[19,36]
2	Dosing frequency	Administered at intervals (weekly, fortnightly, three-weekly)	Daily, every alternate day, weekly	[19,23]
3	concentration of plasma	Shifted	Maintained	[19,23,53]
4	Cells targeted	Dividing cancer cells	Angiogenically stimulated endothelial cells in the progressing vasculature of the tumor	[19,54]
5	Purpose of administration	To treat cancer directly by inhibiting or eliminating rapidly proliferating tumor cells	To achieve cancer control by focusing on angiogenesis	[19,23]
6	Toxicity	Toxicity is a significant issue as a result of drugs used at maximum tolerated doses	No notable toxic side effects were observed due to the achievement of a continuous low level of drugs in the bloodstream	[19,36]
7	Effectiveness	Much effective in the primary tumor than in metastasis	Advanced cancer	[19,23]
8	Quality of life	Poor	Improved	[50]

## 4 Mechanisms of metronomic action

Metronomic chemotherapy both directly and indirectly affects cancer cells and their surrounding hence is a therapy with multiple targets. It can impede tumor angiogenesis, enhance the immune system's ability to fight tumors, and even cause tumor dormancy [19]. Previously MCT was shown to have antiangiogenic activity, however, quite recently it has been thought to improve its antitumor effects through several interconnected mechanisms, which differentiates it from traditional chemotherapy [32].

### 4.1 Metronomic chemotherapy enhances the antiangiogenic activity

The vascularization process is mediated by a well-regulated sequence of events involving the release of the growth factors, both pro‐angiogenesis, and anti‐angiogenesis, from platelets. Additionally, circulating micro-particles derived from megakaryocytes, which are involved in wound healing, play a role. Platelets release the initial pro‐angiogenesis regulators, such as vascular endothelial growth factor (VEGF), triggering the initiation of vessel tip formation during wound healing [55,56]. The next step is the release of growth factors that are necessary for vessel stalk development and bFGF and PDGF are two of these components [56,57]. Angiogenesis inhibitors are finally released from the platelets, which leads to the death of proliferative cells, vessel pruning, and the cessation of angiogenesis [49,58].

VEGF, the most potent and well-known angiogenic activators, induces neo-vascular networks and tumor angiogenesis that provide tumor cells with oxygen and nutrients, resulting in tumor progression, invasion, and metastasis [59]. The sequential steps of tumor angiogenesis typically involve activation, proliferation, and migration of endothelial cells found in the normal blood vessels, and this process is termed tumor sprouting angiogenesis. According to this concept, the treatments against angiogenesis targeting the VEGF/VEGF receptor (VEGFR) pathway may prevent the formation of tumor vessels, halting the spread of cancer and its metastases [60]. Patients with different types of advanced malignancies are currently treated with a variety of humanized monoclonal antibodies (such as bevacizumab and ramucirumab) and small molecules (such as sunitinib and axitinib). These therapeutic agents effectively block tumor angiogenesis by specifically targeting endothelial cells associated with the tumor [61,62].

The progression and metastasis of malignant tumors depend on angiogenesis, or the formation of new blood vessels. Non-cancerous cells, in particular endothelial cells, may survive the standard MTD schedule of cytotoxic drugs during the drug-free intervals, resulting in neo-angiogenesis, tumor progression, and ultimately metastasis. Clinical studies have demonstrated that metronomic chemotherapy (MCT) when administered on a low-dose, regular regimen has an antiangiogenic impact [17,41,63].

Endothelial cells in the tumor neovasculature can undergo apoptosis or growth arrest in one of two ways as a result of metronomic chemotherapy [17]. According to a “direct” mechanism, activated, differentiated endothelium cells are inherently vulnerable to low-dose chemotherapy, which is supported by some research. Circulating endothelial progenitor cells may also be in this category. The “indirect” method makes the assumption that endothelial cells won't undergo growth arrest or apoptosis because the drug levels being metronomically delivered are too low. Instead, low-dose chemotherapy causes some cells to produce thrombospondin 1, an endogenous inhibitor of angiogenesis. Without any negative side effects like myelosuppression, hair loss, nausea, or vomiting, this reduces tumor neovascularization by inhibiting tumor angiogenesis and vasculogenesis [64,65].

### 4.2 Metronomic Chemotherapy Promotes Anti Tumor Immunity

The immune system plays a critical role in the emergence and management of cancer. Neutropenia and lymphopenia are two of the most notable side effects of chemotherapy on the immune system, although numerous research has shown and reviewed that some cytotoxic medicines, including anthracyclines, taxanes, and cyclophosphamide, have immune-stimulating qualities [66]. However, the influence of MCT on regulatory T cells (Tregs) is significant in this regard. Tregs (CD4^+^ CD25^+^ Foxp3+ cells) can suppress the immune system's response (antigen-specific) in both cytokine- and cell-contact-dependent manner [67]. Regulatory T cells can thereby prevent the anti-tumor immune response by inhibiting the function of both tumor-specific effector cells (natural killer cells and NK T cells) and tumor-unspecific effector cells (CD8^+^ cytotoxic T lymphocytes and CD4^+^ T helper cells). Treg cells have been discovered in higher concentrations in different human malignancies, which may be related to tumor development and poor response to treatment [[67], [68], [69]]. Therefore, reducing Treg activity through either targeted inhibition or depletion is a way to improve the immune response against tumor-associated antigens. The impact of minimal-dose cyclophosphamide on Treg cells has been studied extensively (both in preclinical and clinical settings). It enhances memory T cells and lymphocyte proliferation while decreasing the amount of Treg cells and suppressing their function [67,70].

High-dose chemotherapy can impair the immune system function because cytotoxic drugs destroy immune cells, preventing them from pursuing therapy-resistant cancer cells. However, the low-dose chemotherapy that is given on a regular schedule can enhance the destruction of immune-suppressive regulatory T cells (Tregs) [[71], [72], [73]], boost dendritic cell activity [74], encourage the maturation of antigen-presenting cells (APCs) [75], and most importantly, enhance the activation and functionality of cytotoxic NK and CD8^+^ T cells [[76], [77], [78]]. Tanaka et al. discovered a class of drugs that caused DC maturation after classifying and evaluating chemotherapeutic drugs according to their impact on DCs. Vinblastine specifically boosted the maturation of DCs at low doses, as seen by the increased expression of markers such as CD80, CD86, MHC-II, and CD40. Vinblastine is extremely suppressive of anti-tumor immunity at high concentrations [75]. Another study conducted found that, vinblastine boosted the activity of cytotoxic lymphocytes against mouse B16 melanoma targets; preventing the B16 melanoma from growing as it would otherwise [75,79]. Specifically, a study conducted by Doloffand and Waxman showed that NK cells, DC cells, and macrophages were significantly recruited and activated as a result of metronomic cyclophosphamide (CTX) injection every six days (Q6day cycle). An abrupt regression of the implanted glioma xenografts coincided with this response. The mechanism depends on the VEGFR2 receptor's concurrent active signaling [80]. Cyclophosphamide treatment when given more frequently caused serious harm to the NK cells themselves, while treatment that was given less frequently—Q9day and Q12day schedules—led to tumor escape. The CD8^+^ T cells increasing activation and functioning showed similar results [77]. The study also demonstrated that by activating and safeguarding anti-tumor immune responses, establishing the right time and dose schedule may significantly improve treatment outcomes. Future research will need to address the problem of finding such a combination for every form of cancer.

Furthermore, metronomic chemotherapeutic drugs have an impact on myeloid-derived suppressor cells (MDSCs) as well, which mitigates the suppression of adaptive immune responses and enhances anti-tumor activity. MDSCs are known for their immature status and capacity to inhibit T cells and comprise a diverse group of immune cells of the myeloid lineage [81]. Research conducted by High fill and colleagues [82] showed that the aggregation of suppressor cells of myeloid origin in the tumor bed can reduce the effectiveness of checkpoint blockade in cancer. But chemotherapeutic drugs like gemcitabine, cluster in, and docetaxel as well as a combination of chemo-immunotherapies that reduce immune cell suppression and encourage anti-tumor responses can effectively target MDSCs [79,83,84].

### 4.3 Metronomic Chemotherapy decreases Therapeutic Resistance

**Fig. 3:**
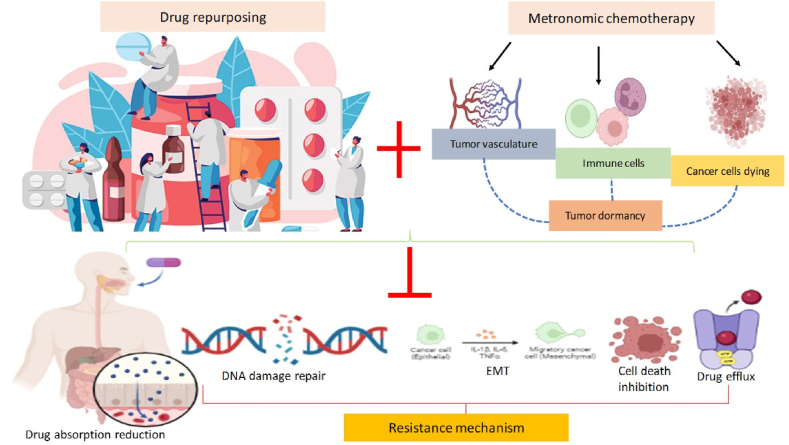
Metronomic chemotherapy in combination with repurposed drugs decreases therapeutic resistance by activating several anti-resistance mechanisms.

To generate the blood vessels required for bringing in nutrients and oxygen, cancer cells rely on the stroma that supports them to give pro-angiogenic signaling [85,86]. But stromal cells, like pericytes and fibroblasts, can be more vulnerable to the effects of chemotherapeutic drugs and can also be harmed by doses that are not toxic to tumor cells. The supply compartment that nourishes both sensitive and resistant cells is equally damaged if the tumor stroma is targeted [34,87]. Particularly when combined with other treatment techniques, this can impair the total tumor population without favouring resistant clones (Fig. 3) [41,42,76,[88], [89], [90]]. Notably, a typical objection against the administration of chemotherapy drugs at low doses is based on a comparison to the ineffectiveness of antibiotics in treating bacterial illnesses [91]. The crucial distinction is that while MCT aims to target not the tumor cells but the stroma that supports them, antibiotics, at any dose, directly target bacteria. As a result, MCT delivery is generally unaffected by the mechanisms that lead to the emergence of antibiotic resistance [86].

### 4.4 Metronomic chemotherapy initiates Tumor Dormancy

Cell-cycle disruption can cause tumor dormancy, or it can result from a powerful balanced scenario where the start of apoptosis balances cell growth[92]. Both during the onset of cancer formation and throughout the remission stage following the completion of anticancer therapy, tumors might recommence their progression from persisting residual illness [93]. Three key mechanisms by which MCT promotes tumor dormancy are immune surveillance, angiogenesis suppression, and programmed cell death of malignant cells [36]. Immune surveillance enables the immune system to monitor, recognize, and eliminate nascent tumor cells. The suppression of angiogenesis, that is the inhibition of blood vessel formation leads to the inhibition of the tumor growth and the apoptotic pathway activation by various mechanisms of MCT leads ultimately to the tumor cell death.

## 5 Unravelling the Role of Low Dose MCT in Different Cancers

Numerous investigations have so far shown that the majority of MCT-based clinical trials were tolerably tolerated. Additionally, as evaluated in multiple clinical trials, this therapeutic approach is regarded as a cost-benefit treatment choice. The severe toxic consequences are either hardly ever observed or extremely uncommon. In general, side symptoms included nausea, minor-grade fatigue, mild to severe leucopenia, lymphopenia, neutropenia, and anaemia. Additionally, MCT offers a remarkable clinical improvement, including an improvement in QOL with minimal toxicity. Here, we've covered recent clinical research on MCT, focusing primarily on lung, colorectal, prostate, gastric, breast, and pancreatic cancers (Table 2) [22].

**Table 2:** MCT for different types of cancers [19,22].

MCT Regimens	Study endpoints	Type of cancer
Irinotecan or fluorouracil (1500 or 2000 mg) was given daily with or without other treatments.	Median Overall Survival of eight months and disease control rate was twenty-six percent, respectively	CC
CPB (1500 mg) was given every d	A low toxicity profile and had not any grade of adverse effects when given a metronomic regimen CPB	CC
VNR (50, 40, and 30 mg) was given orally thrice a week.	With an overall illness control rate of 61.9 % and a response rate of 17.8 %, respectively, VNR showed disease stabilization activity for a long time.	NSCLC
Docetaxel (25 mg/m^2^) was given every week and Trofosfamide (50 mg) was given once every day.	The median OS, OR, and PFS were 9.6 months, nineteen percent, and 2.9 months.	NSCLC
ETP (100 mg) was given orally.	The comparable numbers for stable illness and partial response were thirty-four percent and twenty-eight percent, respectively, along with a median OS and PFS of nine months and 6 months respectively.	NSCLC
MTX (9 mg/m^2^) was given to patients with head and neck cancer.	The OS rate and PFS were six months, and three months respectively along with an OR rate of 42.9%.	HNC
MTX (15 mg/m2) was given orally once weekly + Celecoxib (200 mg) was given two times every day, or Cisplatin (75 mg/m^2^) was given once every 3 weeks for six cycles.	The median overall survival was 7.5 months when given metronomic regimens. When given Cisplatin the median OS was 6.1 months.	**HNC**

Abbreviations: CC: Colorectal Cancer; NSCLC: Non-small cell lung cancer; HNC: Head and Neck Cancer; VNR: Vinorelbine; CPB: Capecitabine; MTX: Methotrexate; ETP: Etoposide; OS: Overall survival; OR: Overall response; PFS: progression-free survival.

### 5.1 Non-small cell lung cancer (NSCLC)

With about 20 % of all cancer-related deaths occurring from lung cancer, it is also one of the most common cancers worldwide [94]. Although targeted and immunological therapies have advanced, chemotherapy is still frequently given to lung cancer patients during their oncological condition [95,96]. Due to its toxicity, traditional chemotherapy may not be a good solution for elderly or disabled people. MCT, an effective and better-tolerated regimen for this subtype sufferer, can solve this issue. The antineoplastic drug VRB, administered orally as a monotherapy, has been the subject of the most phase II research for non-small cell lung cancer (NSCLC). For patients deemed unsuitable for platinum-based treatments or who were already getting multiple medications, the most popular schedule was monotherapy, administered three times per week. With an overall DCR and RR of 61.9% and 17.8%, respectively, the data from a multicenter international retrospective examination on 270 NSCLC individuals who received MCT VRB via oral route at MCT doses of 50 mg, 40 mg, and 30 mg thrice a week as 1st, 2nd, or subsequent line, respectively, depicting disease stabilization activity for a longer time [97]. Another study looked at 30 mg of oral VRB given thrice a week to chemotherapy patients and found that the median PFS was four months, the ORR was 26%, and the median OR was seven months [98]. In 62 patients with IV NSCLC, Goern et al. utilized a weekly schedule of 25 mg/m2 DTX and 50 mg trofosfamide once daily. The median OS, overall response, and PFS in this research, respectively, were 9.6 months, 19%, and 2.9 months [99]. Patients with NSCLC received 100 mg of oral ETP as part of another research. In addition to median OS of nine months and TTP of six months, the equivalent numbers for stable disease and partial response were thirty percent and twenty eight percent, respectively [100]. Bevacizumab was combined with a metronomic schedule of gemcitabine and PCL, along with alternative evidence of vascularization. For 6 and 12 months, respectively, the median PFS rates for 39 patients with advanced NSCLC were seen to be 61 % and 21 %, while the ORR and median OS were both 56 % [101].

### 5.2 Colorectal cancer (CC)

Additionally, CC is among the most common malignancies and a major global cause of cancer-related death [102]. The foundation of the best chemotherapy for colorectal cancer is fluoropyrimidines. They are acknowledged as the main treatment for metastatic colorectal cancer until new anticancer medications like OXA and irinotecan and biological medicines like cetuximab, bevacizumab, and panitumumab are approved [22]. Recently, the treatment alternatives regorafenib and trifluridine/tipiracil (TAS-102) for extensively researched metastatic CC were approved. These therapeutic strategies, however, might not be appropriate for some elderly individuals or individuals patients who have undergone extensive treatment, as disease management with a high safety profile and improved QOL is often required. According to numerous studies, MCT is an effective and secure treatment option for this particular group of patients. Additionally, other anticancer medication classes were looked at for CC as MCT. These drugs are irinotecan, CPB, and cyclophosphamide; nevertheless, the oral delivery of CPB had the best results. A consistently prescribed daily dose of CPB has been considered a therapy option for colorectal cancer since 1998. The administration of irinotecan or fluorouracil (1500 mg or 2000 mg daily) on a continuous schedule to 50 patients with or without other therapies resulted in a mild toxicity profile, and none of the patients receiving CPB on a metronomic schedule exhibited any grade of adverse effects [103]. Furthermore, a more recent retrospective analysis of 68 patients who had previously received treatment with a metronomic regimen of CPB at a daily dose of 1500 mg revealed median OS and disease control rates of eightmonths and twenty six percent, respectively [104]. Moreover, a randomized phase III clinical trial showed that maintenance therapy and a metronomic schedule of CPB plus bevacizumab administered after 6 cycles of conventional CPB + bevacizumab + OXA significantly extended PFS compared to the monitoring group (11.7 vs. 8.5 months) without degrading quality of life [105]. These results emphasize the efficacy of metronomic CPB as a treatment option in elderly and extensively pretreated metastatic cancer; nevertheless, further research is needed to assess its efficacy as maintenance therapy.

### 5.3 Head and Neck Cancer (HNC)

Recurrent, relapsed, or newly diagnosed HNC that are not responsive to any targeted therapy upfront benefit greatly from palliative systemic therapy. Due to their high cost, the lack of regimens for the palliation of individuals with HNC remains a significant social and ethical issue in low and middle-income countries (LMICs) [106]. Patil et al. enrolled the first 15 patients in a phase I-II research and determined that 9 mg/m2 of MTX was the ideal biologic dose (OBD). Additional 91 patients were enrolled in the Phase II portion of the same study; the 3-month PFS rate was 71.1% (95 % confidence interval [CI], 60.5% to 79.3%), the 6-month OS rate was 61.2% (95% CI, 49.2% to 67.8%), and the ORR was 42.9% (95% CI, 33.2% to 53.1%; n = 39) [107]. Adult individuals aged 18–70 years who were already eligible for palliative systemic therapy for relapsed, recurrent, or newly diagnosed SCHNC were enrolled in a subsequent randomized Phase 3 trial [108]. They were randomly assigned to receive either oral MCT (MTX 15 mg/m2 once per week + Celecoxib 200 mg twice per day, or Cisplatin 75 mg/m2 once every three weeks for 6 cycles). Even though intravenous cisplatin is the standard of therapy in LMICs, patients who got MCT had better results than those who received it. In the MCT group, the median OS was 7.5 months (IQR 4.6–12.6) while it was 6.1 months (3.2–9.6) in the cisplatin group. 19 % of the group receiving MCT had grade 3 or higher toxicity, compared to 30 % of the group receiving conventional treatment (p = 0.01). Even though there are some serious concerns with these findings, this study provides a good illustration of how to strike a compromise between the need to treat patients in LMICs and the need to maintain the effectiveness of the treatment.

### 5.4 Breast cancer

Several MCT clinical studies have been completed in recent years, highlighting the expanding acceptance of this method of chemotherapeutic delivery in various malignancy types. There have already been six separate meta-analyses published thus far, mostly focusing on glioblastoma [109], lung cancer [96,110], and breast cancer. One of the malignancies for MCT that is most likely to be researched is breast cancer [51,111]. The most frequently used chemotherapeutic drugs examined in metronomic studies include CTX, MTX, vinorelbine (VNR), and CPB. CPB and VNR have undergone the most comprehensive evaluations as single agents [22,112]. Taguchi et al. in 2010 examined the effectiveness of low doses of CPB as first-line therapy in 33 patients with metastatic BC. The following schedule for continuous administration of capecitabine was used: For 21, 825 mg/m^2^ was given twice daily out of twenty-eight days. The results of this investigation showed that CPB was effective and well tolerated in this regimen with overall survival (OS) and progression-free survival (PFS) median of 6.9 and 24.8 months [113]. Another phase II trial examined the effects of three distinct treatment plans on 323 patients with metastatic BC. The drug schedule for the study used was: 1000 mg/m^2^ of CPB was given twice daily for fourteen of every twenty-one days, 650 mg/m^2^ of CPB twice daily regularly or conventional CMF (a combination of support/facing-breast-cancer/going-through-treatment-breast-cancer/chemotherapy" title="https://breastcancernow.org/information-support/facing-breast-cancer/going-through-treatment-breast-cancer/chemotherapy">chemotherapychemotherapeutic drugs namely cyclophosphamide, methotrexate, and 5-fluorouracil). Both CPB and conventional administration of CMF schedules had shown fewer adverse effects and were effective [114].

In 60 patients with metastatic BC who had already received treatment, Fedele et al. assessed the effectiveness of continuing CPB at a dose of 1500 mg once a day. According to the study, the median time to progression (TTP) was 7, the median OS was 17 months and the clinical benefit rate (CBR) was 62 % [115]. VNR dose of 70 mg/m^2^ to 34 older patients was subjected three times weekly, followed by a one-week break. With a median PFS of 7.7 and OS of 15.9 months, respectively, this metronomic regimen was well tolerated with a grade 3 neutropenia at 6 % [116].

Another study conducted by De Iuliis and colleagues investigated VBR in 32 individuals at a dose of 30 mg administered orally every alternate day. A 50 % clinical benefit rate and an ideal safety profile (no incidents of grade 3 or 4) were reported [117].

MCT has been used in many clinical trials, most of which were phase II trials, on adult patients with BC, lung carcinoma, prostate carcinoma, malignant brain tumors, colon carcinoma, multiple melanomas, malignant lymphoma, Hepatocellular carcinoma, including other forms of tumors [[118], [119], [120]]. Chemotherapeutic and antiangiogenic drugs were used in many of these clinical trials. Metronomic chemotherapy is effective in about 80 % of trials. Orlando and colleagues in their study found that, among patients with advanced BC who had previously developed resistance to trastuzumab, twenty-seven percent of them responded to treatment when a combination of doublet metronomic CTX and methotrexate (MTX) in combination with trastuzumab (TZM) was used. In addition, the positive response was observed not only in terms of improved therapeutic response rates (including complete response + partial response) but also in terms of clinical benefit, which encompasses complete response, partial response, and stable disease [121]. Furthermore, in separate randomized phase III adjuvant trials involving patients with non-small cell lung cancer and BC, Kato et al. and Watanabe et al. respectively found that continuous daily administration of non-toxic doses of UFT (uracil and tegafur) as postoperative adjuvant treatment was both effective and safe [122,123]. UFT can be considered a metronomic chemotherapy-like trial as it was given every day for two years without a break. Fewer clinical trials with metronomic chemotherapy, in contrast, showed unfavourable results [31,[124], [125], [126]].

### 5.5 Role of metronomic chemotherapy in HER2-Positive breast cancer- a subtype of BC

The Human Epidermal Growth Factor Receptor 2 (HER2) gene is amplified or overexpressed in around twenty to twenty-five percent of BC patients, which is linked with a higher risk of recurrence and a poor prognosis [127]. The development of therapies targeting HER2, such as TZM and pertuzumab, benefits patients with HER2+ BC [128,129].

Anti-HER2 monoclonal antibodies may be a straightforward substitute for combination with antiangiogenic therapeutics like mCHT as pre-clinical research suggests they can reduce angiogenesis and tumor growth by controlling pro- and antiangiogenic factors [130,131].

In the mouse model, it was also observed that increased angiogenesis via VEGF overexpression contributed to carcinoma resistance to trastuzumab. The authors further speculated that MCT might prevent or reverse trastuzumab resistance in women with HER2+ BC [132]. Francia and colleagues preclinical trial served as the earliest validation of the longer survival and lessened toxicity of metastatic carcinoma in the setting of trastuzumab combined with metronomic CTX [133]. The administration of metronomic chemotherapy in combination with HER2-targeted therapeutics in metastatic settings was investigated in various trials (phase II), providing a dependable alternative for second-line therapy. A significant portion of patients who developed TZM resistance experienced the therapeutic benefits of TZM when combined with a dual metronomic regimen that included MTX and CTX. This combination has demonstrated excellent effectiveness in treating HER2+ metastatic BC [121].

Orlando et al. conducted a phase II trial of the Gruppo Oncologico Italia Meridionale (GOIM) to investigate the viability of this approach as the first-line therapy for advanced HER2+ BC. They administered a combination of TZM with metronomic CPB and CTX, to patients experiencing their first relapse or with synchronous metastasis. The results demonstrated promising clinical efficacy and remarkable tolerance to the treatment. A clinical benefit rate (CBR) of 78.2 % and an objective response rate (ORR) of 56.7 % (95 % CI, 44.1–68.4 %) were reported [134].

The trial named EORTC 75111- 10,114 enrolled eighty patients diagnosed with metastatic HER2+ BC and were treated with a metronomic oral CTX (50 mg Qd) and dual anti-HER2 agents (trastuzumab + pertuzumab) as first-line therapy. The combination of these treatments showed a significant improvement with 27.2 % increase in median progression-free survival when compared to dual anti-HER2 therapy alone. Moreover, the treatment exhibited a manageable safety profile [135].

Wang and colleagues conducted a single-arm phase II clinical trial to validate the effectiveness of combination treatment of the TZM and metronomic VNR in patients with HER2+ metastatic BC. The study confirmed that the treatment demonstrated both effective anticancer properties and tolerable toxicity in this patient population and also determined that the ORR was 20.0 % and the CBR was 75.0 % [136].

Researchers have investigated the potential of combining metronomic CBP with tyrosine kinase inhibitors (TKIs), in addition to anti-HER2 monoclonal antibodies. A study conducted by Saura and colleagues explored the use of neratinib (a powerful irreversible pan-TKI) in combination with metronomic CBP. The results showed a median PFS of 40.3 weeks in HER2+ BC patients who had not been previously treated with lapatinib and 35.9 weeks in patients who had already received lapatinib [137].

Additionally, MCT's effectiveness and safety in the neoadjuvant treatment of BC are being gradually confirmed. In the TraQme phase II clinical trial, individuals with locally advanced stage III HER2+ BC who had not received prior treatment were enrolled. The trial employed a combination therapy of doxorubicin, TZM, and metronomic CTX, followed by paclitaxel and TZM in the pre-operative treatment regimens. The pathologic complete response (pCR) rate was observed to be 55.5 %, with a median follow-up period of 33.6 months [138].

A clinical trial named NCT01873833 investigated the combination of TZM with metronomic drugs (CAPE + CTX + Lapatinib) in patients with HER2+ metastatic BC who had previously received TZM administered in either the metastatic or adjuvant setting. Pyrotinib, a recently created TKI in China, has now the second-line standard treatment for HER2+ metastatic BC due to its success when combined with CBP [139]. To further explore treatment options, Li et al. conducted a single-arm, interventional, phase II clinical trial (NCT03923166) to examine the effectiveness and security of erlotinib combined with metronomic CBP in HER2+ metastatic BC [140].

#### 5.5.1 Triple-negative breast cancer (TNBC)- an aggressive subtype of BC and its response to metronomic chemotherapy

About fifteen percent of all cases of BC fall under the category of TNBC, which is distinguished by highly violent, early recurrence, a poor prognosis, and few treatment options. TNBC exhibits a distinct molecular composition, characterized by overexpression of epidermal growth factor receptor (EGFR) and VEGF, which is implicated in the angiogenesis modulation, in place of the expression of estrogen receptor (ER), progesterone receptor (PR), and HER2. As a result, there is mounting proof that TNBC patients can benefit from anti-angiogenic therapy. It had previously been clarified that their antiangiogenic effectiveness might be increased when combined with a metronomic schedule [141].

Pre-clinical research suggests that the concomitant administration of an angiogenic TKI inhibitor and continuous low-dose metronomic topotecan may have therapeutic benefits for metastasizing TNBC patients [142]. Erlotinib (recently explored small-molecule tyrosine kinase inhibitor), and bevacizumab (humanized monoclonal antibody against VEGF) showed activity in metastatic breast cancer that had already received treatment [143].

In patients with HER2-negative and low hormone receptors (HRs) expression metastatic BC, Montagna, and colleagues conducted a phase II clinical trial combining MCT (CTX + CPB) with antiangiogenic bevacizumab with erlotinib. With a median therapeutic benefit of seventy-five percent and a median time to progression (TTP) of forty-three weeks, the combination treatment was effectively administered and well tolerated [144].

Immunotherapy has been a promising field of oncology in recent years [145]. Although metastatic TNBC does not have a specific targeted therapy, unlike other BC subtypes, it is a desirable target for immunotherapy due to its generally greater amount of TILs and immune checkpoint molecules [146,147]. Due to the variable tumor immune microenvironments, the PD-1/PD-L1 inhibition produced prolonged responses in metastatic triple-negative BC; however, only a percentage of patients benefited from the immunotherapy [148,149].

In metastatic TNBC, PD-1/PD-L1 inhibition produced prolonged responses; however, only a portion of patients benefited from immunotherapy due to diverse tumor immune microenvironments. In the TONIC trial, short-term or MCT may create an immune milieu that is more favorable and increase the therapeutic responsiveness to PD-1 blocking in metastatic triple-negative BC [150].

In two examples of patients with recurrent metastatic TNBC, Chue, and colleagues described the use of sequential MCT in conjunction with immunotherapy. Both of these patients achieved extended remission, creating a potential treatment alternative [151]. Additionally, pre-clinical TNBC mouse models were treated with a combination of metronomic paclitaxel and PD-1 monoclonal antibody (mAb), and this resulted in a potent anticancer effect [152]. This study also put forth the idea that metronomic paclitaxel improved immunotherapy potency by altering the immunological milieu. Toripalimab, an anti-PD-1 antibody, is being tested in a single-arm, clinical trial (phase II) named NCT04389073 in patients with metastatic, HER2-negative BC to see how well it works clinically when combined with metronomic VNR. They also investigated metronomic chemotherapy in combination with immunotherapy and anti-angiogenesis, the results of which are worth waiting for. 22 clinical studies with roughly 1360 people were analyzed recently in a meta-analysis. The use of MCT in patients with advanced BC has shown good results [153]. The use of MCT has also been studied in the context of early BC, primarily in TNBC patients, and as a follow-up therapy following adjuvant therapy [33,154].

Metronomic MTX for twelve months following adjuvant therapy with carboplatin demonstrated an improved overall survival (OS) in phase III research by Nasr and colleagues [155]. A recent trial (phase III), called SYSUCC-001, examined the effectiveness of metronomic CPB as maintenance therapy for one year following standard curative treatment and found evidence of a considerable improvement in disease-free survival of 83 % compared to 73 % at five years [156].

The combination of MCT with other treatment methods is giving rise to a promising shift in the treatment of cancer, leading to the transformation of carcinoma into a more manageable chronic condition with a range of new therapeutic options [157] (Table 3).

**Table 3:** Metronomic chemotherapy in advanced or metastatic breast cancer (first, second-line chemotherapy).

Breast cancer	Trial name	Protocol	Study design	Study endpoints	References
BC	EudraCT 2010-023,794-19 (Victor 2 study) completed	mVNR-CPB	II	I: Clinical benefit II: Progression-free survival, time to progression, Toxicity	[104]
HER2 (+) BC Elderly	NCT01597414 EudraCT 2011-006342-32	pertuzumab-trastuzumab pertuzumab-trastuzumab-mCTX	II R	I: Progression-free survival II: overall survival, overall response rate, quality of life	[104]
HER2 (−) BC	NCT01941771 EudraCT 2011-003564-72	VNR-CPB mVNR-CPB	II R	I: overall response rate II: time to progression, overall survival, toxicity	[104,158]
BC	NCT01526499	TXT-mCTX TXT	II R	I: progression-free survival II: Overall survival, overall response rate, safety	[104]
HER 2 (−) BC	NCT01131195	BEV-PTX BEV-mCTX-mCPB	III	I: adverse events II: overall response rate, overall survival, progression-free survival	[104]

Abbreviations: BC: Breast cancer; VNR: Vinorelbine; CPB: Capecitabine; TXT: Taxotere; CTX: Cyclophosphamide; PTX: Paclitaxel; BEV: Bevacizumab.

MCT offers a remarkable clinical improvement, including an improvement in quality of life with minimal toxicity. However, to increase the usage of MCT in clinical settings, a well-conducted phase III trial demonstrating the significant value of metronomic dosage is required. Furthermore, well-reviewed randomized phase II studies would also produce strong proof to support the use of MCT. So far, finding clinical trials with a randomized design and statistical significance is still challenging [159,160].

## 6 Metronomic chemotherapy and drug repurposing

As discussed, earlier Metronomics is the combination of MCT and drug repurposing. MCT is based on administering chemotherapeutics on a regular schedule at doses lower than the MTD, with non-prolonged drug-free pauses. Drug repurposing involves employing already-approved medications for whose antitumor characteristics have been identified, leading to new medicinal uses. These medications can be found on the WHO essential medicine list and are generally accessible [29]. Numerous researchers have proposed it as an alternative method to expand the therapeutic options for the treatment of cancer offering numerous benefits over the creation of new cancer drugs. Since the pharmacokinetic and pharmacodynamic properties have already been extensively studied, there is no need for additional investigations, which speed up the translational process and decrease related costs, making the approach a great success [[161], [162], [163]].

At present, as outlined by Kaushik et al. this approach involves some crucial steps that researchers must carry out to determine the repurposing potential of a particular drug [164]. These steps include clinical observations and epidemiological studies in addition to pre-clinical methods like in silico, in vitro, or in vivo studies [165]. Therefore, it is feasible to locate viable candidates for medication repurposing for anti-cancer therapy by identifying the anti-tumor properties of medicine and researching its targeted molecular pathways. Repurposed drugs often exhibit “off-target” effects, which can unexpectedly exhibit anti-tumor properties. These molecular mechanisms interact with any pathway that is connected to the cancer hallmarks proposed by Hanahan in 2000 (updated in 2021) [166,167].

Drug repurposing treatment has its share of difficulties as well [168[169]]. Drugs that have been repurposed can be used alone, as chemopreventive agents, or to complement the effects of other chemotherapeutics. Additionally, they can be used as adjuvant therapy to prevent tumor recurrence or to manage the side effects of other medications. They can be coupled with other medications like metronomic chemotherapy to focus on other oncogenic pathways or to work in concert to completely eradicate the tumor. The likelihood of drug resistance growing as a result of their treatment in monotherapy is increased [170,171].

Since each of the therapeutic drugs can operate through different routes and intensify the overall anti-tumor action, medication combinations are often more effective than monotherapy [161,166]. However, despite the greater anti-tumor effects, a multidrug treatment plan might lead to more undesirable side effects, brought on by drug-drug interactions, which can make the treatment less effective [172]. Maintaining patient follow-up and carrying out more thorough molecular tests are crucial to preventing these problems. The variety of reactions in oncological patients is another important obstacle to be addressed in cancer treatment. Drug Repurposing has the benefit to deliver cytotoxics in a metronomic manner. MCT has been used in conjunction with MTD chemotherapy, radiotherapy, target therapy, immunotherapy, and even with drug repositioning, wherein medications like celecoxib and metformin that interfere with the tumor microenvironment are incorporated in combination regimens, with the goal of maximising therapeutic efficacy in addition to their multiple mechanisms of action and low toxicity profile. The tyrosine kinase inhibitors pazopanib, sorafenib, and gefitinib, as well as the monoclonal antibody bevacizumab, are the most often used target agents investigated with MCT because of their antiangiogenic effects [162].

As a result of the variety of patients, the same medicine may be more or less effective, necessitating the use of more specialized care. Furthermore, it's critical to emphasize that the primary cause of cancer resistance mechanisms is malignant heterogeneity [173]. Therefore, there is a crucial need to change the approach to treating these patients, by giving attention to both intra- and inter-tumor heterogeneity, as relying solely on a standardized treatment model may fail to adequately address the genetic diversity present among cancer patients. In addition to have new antitumor medications available that can be utilized for the patient's treatment [174].

**Fig. 4:**
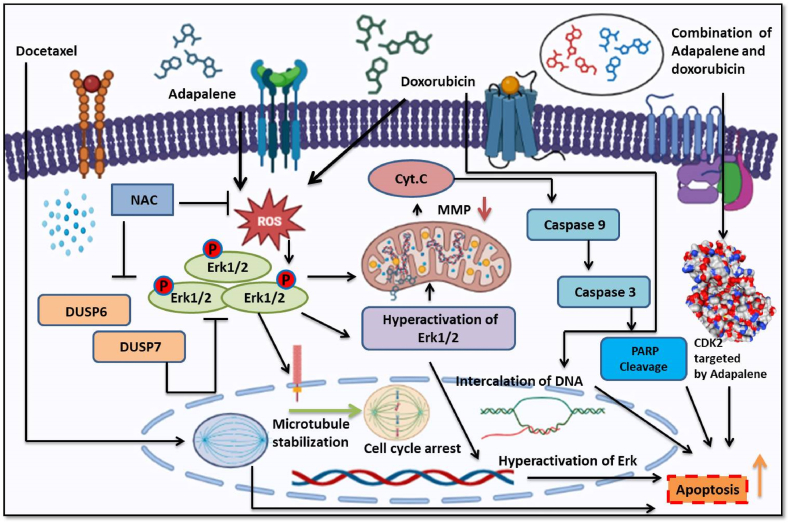
Repurposed drugs used in combination or alone led to increased production of ROS, causing a disturbance in the potential of the mitochondrial membrane. This disturbance in the mitochondrial membrane potential initiated an intrinsic apoptotic process that involved the cleavage of PARP. Additionally, the augmented generation of ROS activated the Erk1/2 signaling pathway, leading to hyperactivation, which further facilitated apoptosis through the mitochondrial death pathway.

With the advancement of genetics, personalized medicine has recently gained attention as experts look for new and effective treatment choices for each patient. Genetic changes in a patient can affect how a drug is absorbed, metabolized, transported, and excreted as well as how it functions in the body (pharmacodynamics), all of which might affect a patient's response to medication [175]. In our lab, we have been working on drug repurposing for the last six years and this approach has shown excellent results in BC. We have deciphered the mechanism of action and repurposing of the drugs such as adapalene, GDC-0941/docetaxel, doxorubicin, etc. In addition, we have used these drugs in combination that have produced fruitful results signifying the importance of drug repurposing either alone or in combination with chemotherapeutic drugs reducing their toxic effects and improving the overall survival of cancer patients represented in Fig. 4 below.

### 6.1 Drug repurposing

In the first place, there is sufficient cumulative evidence from pre-clinical and clinical research to show that metronomic cytotoxics operate differently from the maximal tolerated dose (MTD) method and the MTD toxicity profile of these medicines is also known to oncologists.Lastly, the cost of re-developing these treatments is less likely to impose additional strain on healthcare systems around the world because they are readily available at cheap cost or in generic forms. However, as is evident from the clinical evidence acquired to date, metronomic chemotherapy would not be beneficial to every patient. To find the appropriate setting and patient population to benefit from MCT, whether in mono-or combination therapy, is still to be determined. The oncology community must invest time and effort to ascertain MCT's place in clinical practice [26,176].

### 6.2 Clinical studies on drug repurposing for cancer therapy

Clinical trials focusing on progression-free survival, tumor volume reduction, and other endpoints are examining the efficacy of anti-CSC medicines. The Anticancer Fund is a stand-alone, nonprofit research organisation that conducts investigator-driven clinical studies in an effort to provide patients with new therapy options as soon as feasible. To research and verify the therapeutic efficacy of repurposed medications and combination therapies, they mostly look at phase II and III trials. A combination of two non-cancer medications, one an anti-cholesterol and the other an anti-inflammatory, was examined in a French research called Fluvabrex for Children with Brain Cancer to investigate if this less toxic treatment was effective against low-grade gliomas in children. In 7 of the 10 children, the tumor advancement was stopped for greater than or equal to 6 months, and in 3 of them, it was stopped for greater than or equal to 3 years [177]. The combination of low-dose methotrexate and celecoxib, known as metronomic chemotherapy (MCT), has been shown in another study by Kumar et al. to be a novel treatment that is thought to act by modulating the immune response, inhibiting angiogenesis, and exerting cytotoxic effects, though the precise mechanism of action is unknown. In both curative and palliative contexts, MCT has been found to be quite successful in preventing the progression of tumors in patients with head and neck squamous cell carcinoma. Additionally, it was found that oral metronomic chemotherapy for patients with advanced/recurrent HNSCC that included methotrexate and celecoxib was efficient, well-tolerated, and improved quality of life while having a low toxicity profile [178]. A few more studies are being conducted to look for alternative cancer treatments employing repurposed medications in various cancer types that call for a more toxic chemotherapy and radiotherapy regimen (Table 4).

**Table 4:** Clinical studies for cancer therapy using different repurposed drugs.

S.No.	Study title	Study objective	Cancer type	Trial phase	Status	Outcome	Reference
1	To decrease recurrence, perioperative anti-inflammatory ketorolac was used for breast cancer.	To find out if perioperative ketorolac administration following breast cancer surgery influences survival rates, researchers conducted a prospective, randomized, placebo-controlled, double-blind trial with a two-year inclusion period and a five-year follow-up period.	Breast cancer	III	Completed	Just before breast cancer surgery, a single injection of the NSAID ketorolac had no beneficial effects on disease-free survival.	[177]
2	Decitabine repurposed in Kras-Dependent Refractory Pancreatic Cancer	Decitabine may be used to treat advanced, refractory, KRAS-dependent pancreatic ductal adenocarcinoma (PDAC) in the ORIENTATE trial, a proof-of-concept, biomarker-driven phase-II clinical investigation.	Digestive cancer	II	Recruiting	–	[177]
3	Repurposing of Aspirin in colon cancer for recurrence and survival.	A phase III double-blind, placebo-controlled, randomized investigation evaluating aspirin's effects on colon cancer patients' survival and recurrence (ASPIRIN).	Digestive cancer	III	Recruiting	–	[177]
4	A combination of three repurposed drugs (Clarithromycin - antibiotic, Pioglitazone - anti-diabetes, Treosulfan - a chemotherapeutic drug used at a metronomic dose) after chemotherapy failure in lung cancer	ModuLung: a prospective phase II, randomized multi-center study comparing nivolumab to a bio-modulatory regimen of metronomic low-dose treosulfan, pioglitazone, and clarithromycin in patients with squamous cell lung cancer and non-squamous cell lung cancer, respectively, following platin failure.	Lung cancer	II	Terminated	In other words, compared to the standard treatment, the new treatment did not improve the outcomes of the patients.	[177]
5	Repurposing of angina pectoris medication (nitroglycerin) for lung cancer treatment	A single center non-randomized phase II trial investigating the effect of nitroglycerin on survival in non-small cell lung cancer.	Lung cancer	II	Completed	Adding nitroglycerin to radiotherapy did not improve non-small cell lung cancer survival.	[177]
6	Repurposing of antimalarial drug as a treatment for liver cancer	An investigation on the pharmacokinetics and safety of oral Artesunate (ART) in patients with advanced hepatocellular carcinoma (HCC) was conducted at a single center during the first phase of dosage escalation.	Liver cancer	I	Terminated	–	[177]
7	Using two repurposed medicines and chemotherapy to treat leukaemia patients	AML-ViVA, a randomized, phase II, open-label, multicenter study comparing low-dose azacitidine, all-trans retinoic acid (ATRA), and pioglitazone to the standard dose azacitidine in patients with acute myeloid leukaemia (AML) who are sixty years of age and are unresponsive to standard induction chemotherapy.	Blood cancer	II	Completed	The treatment is safe in this very fragile population, and three out of ten patients achieved a complete response. An additional patient had disease stabilization for 14 months.	[177]
8	NaHCO_3_ for tumor-related pain	This is a single-institution, non-randomized phase I/II trial of NaHCO_3_ for thirty-five patients with moderate to severe tumor-related pain caused by solid metastatic malignancies or hematologic malignancies in conjunction with routine medical care and a stable opioid regimen.	Blood cancer, solid tumors	I/II	Terminated	Due to the poor tolerability of sodium bicarbonate, patients could not comply with the treatment protocol, leading to the project's permanent closure.	[177]
9	Low dose chemotherapy with a combination of 9 repurposed drugs for brain cancer	In a monocentric proof of concept clinical trial, nine repurposed drugs -aprepitant, auranofin, celecoxib, captopril, disulfiram, itraconazole, minocycline, ritonavir, and sertraline were combined with metronomic temozolomide to assess the safety of coordinated undermining of survival pathways in recurrent glioblastoma patients (CUSP9v3).	Brain cancer	I	Completed	Ritonavir, temozolomide, captopril, and itraconazole were the drugs most frequently requiring dose modification or pausing. Progression-free survival at twelve months was fifty percent.	[177]
10	Repurposing of anti-inflammatory and cholesterol inhibitors drugs for children's optic nerve cancer	Fluvastatin and celecoxib were used in a phase I trial to evaluate the safety of the combination in children with refractory optic-pathway glioma (Fluvabrex).	Pediatric brain cancer	I	Completed	The combination of the two drugs showed limited toxicity with preliminary activity in low-grade gliomas.	[177]
11	Treatment of advanced bone cancer with a combination of chemotherapy and immunosuppressants	A clinical trial (phase Ib) of metronomic cyclophosphamide and methotrexate, combined with zoledronic acid and sirolimus, for patients with solid tumors with bone metastasis and advanced, previously treated osteosarcoma (Metzolimos).	Solid tumors Bone cancer	1 b	Completed	The safe dose of sirolimus in combination with the other three drugs was determined.	[177]
12	Curcumin repurposed for endometrial cancer	This was a prospective, monocentric phase II trial aimed at determining if curcumin can reduce inflammatory mediators and immunomodulatory cell types in endometrial cancer.	Gynecological cancer	III	Completed	Curcumin phytosome had modest immunomodulatory effects in endometrial carcinoma and did not significantly improve quality of life.	[177]
13	Vitamin D treatment repurposed for melanoma	This is a double-blind, placebo-controlled phase III trial of high-dose vitamin D3 (cholecalciferol) for patients with stage IB to III cutaneous melanoma.	Melanoma	III	Recruiting	–	[177]
14	The use of a β-blocker (propranolol) before surgery (neoadjuvant) for patients with angiosarcoma.	The aim of the PropAngio study is to evaluate the effectiveness of propranolol monotherapy in patients with angiosarcoma during the neoadjuvant phase.	Sarcoma	II	Recruiting	–	[177]

## 7 Conclusion and future development

Considering the existing experimental, pre-clinical, and clinical evidence, it is worthwhile to develop metronomic chemotherapy as a new therapeutic therapy method to manage a variety of cancer types. Despite an incomplete understanding of the underlying mechanisms, the available data support the potential efficacy of MCT in cancer management. MCT can work on various targets that include endothelial cells circulating endothelial progenitor cells (EPCs), the tumor microenvironment, and the immune system, as opposed to traditional chemotherapy, which solely focuses on tumor cells. Among them, tumor endothelial cells (TECs) and EPCs are specifically highlighted as the primary targets of metronomic chemotherapy.

Consequently, the metronomic chemotherapy regimen successfully prevents tumor angiogenesis, providing cancer patients with improved quality of life due to its little toxicity and minimal side effects. To optimize therapeutic usage, it is crucial to determine the precise molecular targets of TECs in response to metronomic chemotherapy. Due to the current lack of understanding regarding the molecular targets of MCT, the utilization of omics-based pharmacogenetics and pharmacoproteomic techniques become necessary. It should be mentioned that 2nd and 3rd phase studies have shown good results in individuals with a variety of malignancies after MCT. It will soon be necessary to conduct a randomized, double-blind phase III research to precisely assess the potential advantages of MCT.

In our opinion, MCT and drug repurposing is the best combination for treatment which has shown effective results in our lab as our lab is working on drug repurposing for the last six years and these approaches have shown excellent results in breast cancer cell lines MDA-MB-231[179]. The repurposed drugs used are given in low dose metronomics. Also adding to this these approaches in preclinical and clinical studies have shown good efficacy in advanced HER2-positive/HER2-negative and triple-negative breast cancer with less tumor burden and severe organ complications.

Until now, MCT has mainly been limited to the palliative context. However, with the increasing significance of pre-clinical and clinical evidence, it is now appropriate to expand its application to various other areas. Specifically, it should be considered for early-stage breast cancer including other types of cancers. The utilization of oral treatment regimens, which can be administered at home, coupled with their highly favorable safety profiles, could significantly assist in treating patients in countries with limited resources.

## Ethical approval

Ethical approval and consent of patients to participate is not applicable to our study.

**Data availability statement:**The data associated with our work has not been deposited into a publicly available repository. The data will be available on request.

## Funding

This work was funded by the Jammu Kashmir Science Technology & Innovation Council Department of Science and Technology JKST& IC India with grant No. JKST&IC/SRE/885-87.

## CRediT authorship contribution statement

**Nusrat Jan:** Writing – review & editing, Writing – original draft. **Shazia Sofi:** Writing – review & editing, Writing – original draft. **Hina Qayoom:** Writing – review & editing, Data curation. **Aisha Shabir:** Writing – review & editing. **Burhan Ul Haq:** Writing – review & editing, Data curation. **Muzaffar A. Macha:** Writing – review & editing. **Manzoor Ahmad Mir:** Writing – review & editing, Validation, Supervision, Funding acquisition, Formal analysis, Data curation, Conceptualization. **Abdullah Almilaibary:** Formal analysis, Writing – review & editing.

## Declaration of competing interest

The authors declare that they have no known competing financial interests or personal relationships that could have appeared to influence the work reported in this paper.
